# Exercise-related brain remodeling through bidirectional brain-body crosstalk: an integrative narrative review

**DOI:** 10.3389/fnint.2026.1954740

**Published:** 2026-10-14

**Authors:** Dongjing Yin, Cheng Luo, Juanjuan Hu, Shuang-Zhou Bi

**Affiliations:** 1 Department of Physical Education, Chengdu College of University of Electronic Science and Technology of China, Chengdu, China; 2 Physical Education College, Jiangxi Normal University, Nanchang, China

**Keywords:** blood-brain barrier, brain-body crosstalk, cognition, exercise, exerkines, neuroimaging, neuroplasticity, precision exercise

## Abstract

Exercise is associated with favorable brain and cognitive outcomes, yet the supporting evidence spans molecular, cellular, organ, neural-circuit, imaging, and behavioral levels that differ substantially in causal strength. This narrative review develops an integrative framework across biological levels in which exercise-related brain remodeling emerges from bidirectional brain-body crosstalk rather than from a single peripheral factor or a one-way endocrine pathway. The framework links central motor commands, sensory feedback, autonomic regulation, and cerebral hemodynamics with signal generation by skeletal muscle, liver, adipose tissue, bone, the gut microbiome, and the circulating immune-vascular milieu. Metabolites, secreted factors, extracellular vesicles, and neural afferents may influence the central nervous system through blood-brain and blood-cerebrospinal fluid interfaces, circumventricular organs, cerebral endothelium, and brainstem-hypothalamic networks. Candidate central responses include neurotrophic and synaptic signaling, glial-state regulation, adult hippocampal neurogenesis, and neurovascular adaptation, which may correspond to group-level changes in brain structure, functional connectivity, and cognition. However, direct causal mediation across the complete human pathway remains largely untested: many cellular mechanisms derive from animal or *in vitro* studies, whereas human biomarker and neuroimaging findings are often indirect and process-nonspecific. Age, sex and hormonal state, genotype, disease stage, baseline function, exercise dose, recovery, and adherence further shape response heterogeneity. A stronger translational evidence base will require preregistered longitudinal interventions, source-resolved and time-resolved biomarker sampling, cross-species validation, multimodal target-engagement measures, and externally validated prediction models.

## 1 Introduction

Exercise is increasingly recognized as a modifiable influence on brain health across the life course. Its relevance is especially evident in aging, when the maintenance of cognitive function becomes a major public-health priority and the biological burden of neurodegenerative disease increases (Hou et al., 2019; Tari et al., 2025; Li et al., 2025). Exercise is attractive as a non-pharmacological strategy because it can simultaneously alter cardiorespiratory fitness, metabolism, vascular function, immune state, motor competence, and psychosocial engagement. Nevertheless, the biological explanation for exercise-related brain benefit is not a single mechanism. Evidence is distributed across experimental systems and measurement scales: molecular studies emphasize neurotrophic signaling and synaptic proteins; exercise physiology focuses on muscle, liver, adipose tissue, bone, and systemic metabolism; neuroimaging studies quantify gray matter, white matter, perfusion, and large-scale networks; and behavioral studies evaluate cognition, mood, and motor learning. These levels cannot be joined into a causal chain merely because their changes occur after exercise.

Recent work on inter-organ communication provides a productive way to integrate this fragmented literature. Exercise changes the metabolic and secretory states of several peripheral organs and may convey information through circulating molecules, extracellular vesicles, vascular and barrier interfaces, and neural afferents (Wang et al., 2025; Yin et al., 2022; Kong et al., 2025; He et al., 2024; Blume and Royes, 2024). At the same time, the brain is not a passive recipient. Motor commands, autonomic output, neuroendocrine regulation, sensory feedback, and changes in cerebral blood flow are present from the onset of exercise and continuously reshape peripheral responses. The appropriate unit of explanation is therefore a bidirectional network rather than a peripheral-to-brain pipeline.

In this review, “brain remodeling” refers to relatively sustained molecular, cellular, vascular, structural, or functional adaptations associated with repeated exercise exposure and detectable beyond the immediate physiological response to a single bout. This term does not encompass every acute exercise-responsive phenotype. Transient changes in circulating metabolites, cytokines, catecholamines, cerebral perfusion, blood-oxygen-level-dependent signals, arousal, or task performance are treated here as acute physiological or neural responses that may participate in, precede, or modulate longer-term adaptation but are not themselves considered evidence of brain remodeling. Distinguishing these timescales is essential because an acute response can represent a regulatory signal, a temporary state, or a candidate trigger without demonstrating persistent neural adaptation.

Several previous reviews have already advanced exercise–brain research beyond single-molecule explanations, but they have emphasized different parts of the mechanistic landscape. Stillman et al. (2016) organized potential mediators across molecular, brain-systems, and behavioral levels; Yin et al. (2022) examined bidirectional interactions within the brain–gut–muscle axis and their relevance to energy homeostasis; Blume and Royes (2024) focused specifically on exercise-responsive peripheral factors that may signal to the hippocampus; Tari et al. (2025) emphasized endurance exercise, cardiorespiratory fitness, cerebral blood flow, and neuroprotective mechanisms in healthy brain ageing; and Kong et al. (2025) integrated metabolic and neuroimmune communication within the brain–muscle–liver axis in aging and neurodegeneration. Together, these reviews establish the importance of multilevel and inter-organ perspectives, but they differ in the biological axes, directions of communication, populations, and outcomes that they prioritize.

Building on these complementary perspectives, the present review focuses on the intersection of four features that have not typically been foregrounded together. First, it places descending brain-to-body regulation–including motor command, autonomic output, neuroendocrine signaling, and behavior–within the same framework as ascending molecular, vascular, and neural feedback from peripheral tissues. Second, it treats cardiovascular and cerebrovascular regulation, the blood–brain and blood–cerebrospinal fluid interfaces, cerebral endothelium, circumventricular organs, and neural afferents as an explicit mechanistic layer between peripheral signal generation and central responses. Third, it distinguishes transient responses to acute exercise from the more sustained adaptations required to support claims of brain remodeling. Fourth, it separates preclinical mechanistic evidence from human systems-level phenotypes and evaluates cross-level causal linkage explicitly rather than inferring a continuous pathway from parallel findings across experimental levels.

## 2 Literature identification and evidence organization

This narrative review used a structured literature identification approach to improve transparency while maintaining the integrative purpose of the article. Literature was identified through searches of PubMed/MEDLINE, Web of Science Core Collection, and Scopus from database inception to 1 July 2026, supplemented by reference screening of key reviews and influential primary studies. The search concepts were developed around three major domains: exercise exposure (e.g., “exercise,” “physical activity,” “training,” “fitness”), brain-related outcomes (e.g., “brain,” “cognition,” “neuroplasticity,” “neuroimaging”), and mechanisms of brain–body communication (e.g., “myokine,” “exerkine,” “extracellular vesicle,” “blood–brain barrier,” “gut–brain axis,” “neurovascular signaling,” and “autonomic regulation”). English-language publications were considered. Targeted supplementary searches also addressed cross-species exercise standardization using terms such as “rodent exercise intensity,” “lactate threshold,” “gas-exchange threshold,” “VO2max,” and “cross-species translation.”

Eligible evidence included human studies, animal studies, and mechanistic experimental studies that examined exercise-related alterations in brain structure or function, cognition, molecular signaling, peripheral-to-central communication pathways, or central regulation of peripheral responses. No restriction was applied to study design because the objective was not to estimate pooled effects but to integrate evidence across biological levels and identify mechanistic relationships and translational limitations.

Evidence was interpreted according to biological context and level of inference. Human observational and intervention studies were prioritized when evaluating translational relevance, including exercise-induced changes in biomarkers, neuroimaging phenotypes, cerebral physiology, and behavioral outcomes. Animal and cellular studies were considered primarily for evaluating biological plausibility, molecular mechanisms, and pathway-level interactions that cannot be directly examined in humans. The final selection emphasized studies that provided mechanistic insight, represented important advances, or addressed unresolved controversies relevant to the proposed framework.

Qualitative evidence characterization was based on three predefined dimensions: (1) experimental consistency, referring to whether mechanistic findings were reproduced across animal, cellular, or other experimental models; (2) human directness, referring to whether human evidence directly assessed the proposed pathway or relied mainly on circulating biomarkers, imaging phenotypes, or observational associations; and (3) cross-level causal linkage, referring to whether evidence connected the peripheral source, transmission or sensing interface, central target, and downstream human outcome in a temporally ordered pathway. These dimensions were considered separately rather than combined into an overall certainty score.

This review was designed as a narrative integrative synthesis rather than a systematic review. Therefore, it did not aim to provide exhaustive study enumeration, formal risk-of-bias assessment, or quantitative certainty grading. The evidence characterizations presented in Table 1 are intended to clarify inferential boundaries rather than serve as formal evidence ratings.

**TABLE 1:** Major candidate brain-body crosstalk pathways related to exercise.

Peripheral source, system, or communication route	Candidate signals or routes	Transmission or sensing interface	Candidate central responses	Evidence characterization	Main human evidence	Key limitations
Skeletal muscle	IL-6; lactate; irisin/FNDC5; cathepsin B; IL-15; PGC-1alpha1-kynurenine pathway; local BDNF	Circulation; monocarboxylate transporters; cerebral endothelium; indirect immune-vascular signaling; possible neural routes	Metabolic support; BDNF-TrkB and synaptic signaling; neuroimmune modulation; candidate plasticity effects	Experimental: replicated; Human: mainly biomarker-based or associative; Cross-level: incomplete.	Acute or training-related circulating changes and associations with fitness, memory, or metabolic state	Source attribution, assay variability, sampling window, and causal mediation remain uncertain
Liver	Beta-hydroxybutyrate; GPLD1; predominantly hepatic FGF21-related signaling; LEAP2-related signaling	Circulation; monocarboxylate transporters; blood-brain barrier; choroid plexus; circumventricular sensing	Metabolic and epigenetic regulation; candidate neurogenic and neurovascular effects	Experimental: substantial animal-model support; Human: limited direct pathway evidence; Cross-level: incomplete.	Exercise-, fasting-, or metabolic-state-related changes in metabolites and hepatokines	Nutritional-state and dose dependence; limited human source tracking and target validation
Adipose tissue	Adiponectin; leptin; inflammatory mediators; adipose-derived extracellular-vesicle cargo	Endocrine circulation; endothelial or barrier receptors; hypothalamic sensing; possible EV-mediated delivery	Energy-homeostatic and neuroendocrine regulation; inflammatory modulation; candidate neurovascular or neural effects	Experimental: adipokine signaling and adipose-derived EV communication are supported in experimental models; Human: evidence is mainly based on circulating adipokines and associative or intervention-related changes, with tissue-source uncertainty; Cross-level: unresolved.	Training-related adiponectin and leptin changes, often concurrent with altered adiposity or energy balance	Changes in fat mass and energy balance confound exercise effects; circulating markers do not establish adipose origin; depot and cell-type heterogeneity and EV brain targeting remain unresolved
Bone	Osteocalcin and undercarboxylated osteocalcin	Endocrine circulation; putative barrier or neural routes	Candidate effects on neurotransmission, memory, and systemic metabolism	Experimental: animal-model support; Human: limited and inconsistent; Cross-level: unresolved.	Exercise-related changes in circulating osteocalcin forms and cross-sectional associations	Active form, assay comparability, dose-response, and causal significance are inconsistent
Gut microbiome	Short-chain fatty acids; bile acids; tryptophan and indole metabolites	Immune and endocrine pathways; vagal and spinal afferents; barrier and circumventricular interfaces	Neuroimmune modulation; neurotransmitter-related metabolism; brainstem-hypothalamic regulation	Experimental: substantial; Human: heterogeneous and mainly associative; Cross-level: unresolved.	Training-related microbiome and metabolite differences with variable cognitive or mood associations	Diet, adiposity, medication, geography, and sampling confound results; directionality unresolved
Immune-vascular milieu	Exercise-responsive cytokines; endothelial mediators; prostaglandin-related signaling	Cerebral endothelium and neurovascular unit; receptor-mediated barrier signaling	Glial-state regulation; inflammatory modulation; perfusion and vascular adaptation	Experimental: consistent vascular and inflammatory support; Human: biomarker and physiological evidence, with limited pathway specificity; Cross-level: incomplete.	Inflammatory, endothelial, perfusion, and vascular biomarkers change in some interventions	Systemic biomarkers are not brain-specific; source and temporal interpretation remain difficult
Circulating extracellular vesicles (multi-tissue origin)	Proteins; microRNAs; lipids; metabolites packaged in circulating EVs	Circulation; endothelial uptake; possible blood-brain or blood-CSF interface transfer	Distributed delivery to vascular, glial, or neuronal targets	Experimental: EV-mediated cargo delivery and intercellular signaling are well supported; Human: exercise-related changes in circulating EV abundance or cargo are reported, but tissue origin is often unresolved; Cross-level: brain targeting and target-cell engagement remain unresolved.	Exercise-associated changes in circulating EV abundance or cargo profiles	Tissue origin, biodistribution, interface crossing, target-cell entry, and biologically effective dose remain unclear
Neural afferents	Vagal and spinal interoceptive signals from cardiopulmonary, gastrointestinal, hepatic, and immune states	Nucleus tractus solitarius; brainstem-hypothalamic networks; parallel spinal pathways	Autonomic regulation; arousal; neuromodulation; emotional and cognitive context	Experimental: anatomy and physiology established; Human: autonomic and brainstem responses supported; Cross-level: exercise-specific molecular coding incomplete.	Exercise-related autonomic dynamics and brainstem-linked physiological responses	Not all metabolic or inflammatory information is directly encoded by vagal endings

Evidence characterization was based on three predefined dimensions: experimental consistency, human directness, and cross-level causal linkage. “Experimental” refers to mechanistic support from animal, cellular, or other experimental models; “Human” indicates whether the proposed pathway has been examined directly in humans or primarily through biomarkers, imaging, or associations; and “Cross-level linkage” indicates whether the source, interface, central target, and downstream human outcome have been connected within a temporally ordered causal pathway. These descriptors are qualitative and are not formal certainty ratings or evidence grades. BDNF, brain-derived neurotrophic factor; CSF, cerebrospinal fluid; FGF21, fibroblast growth factor 21; FNDC5, fibronectin type III domain-containing protein 5; GPLD1, glycosylphosphatidylinositol-specific phospholipase D1; IL, interleukin.

## 3 Exercise as a bidirectional perturbation of brain-body systems

Exercise represents a dynamic perturbation of the brain–body system in which central regulation and peripheral adaptation occur simultaneously rather than sequentially. At exercise onset, central motor commands, autonomic adjustments, and neuroendocrine responses prepare peripheral systems for anticipated energetic demands, while proprioceptive, cardiopulmonary, and interoceptive feedback continuously updates central networks. In parallel, skeletal muscle contraction, altered blood flow, substrate utilization, mechanical loading, and metabolic stress generate peripheral signals that can influence central function through humoral, vascular, and neural pathways. Therefore, exercise adaptation should be interpreted as a bidirectional communication process rather than a unidirectional peripheral-to-brain cascade (Figure 1).

**FIGURE 1:**
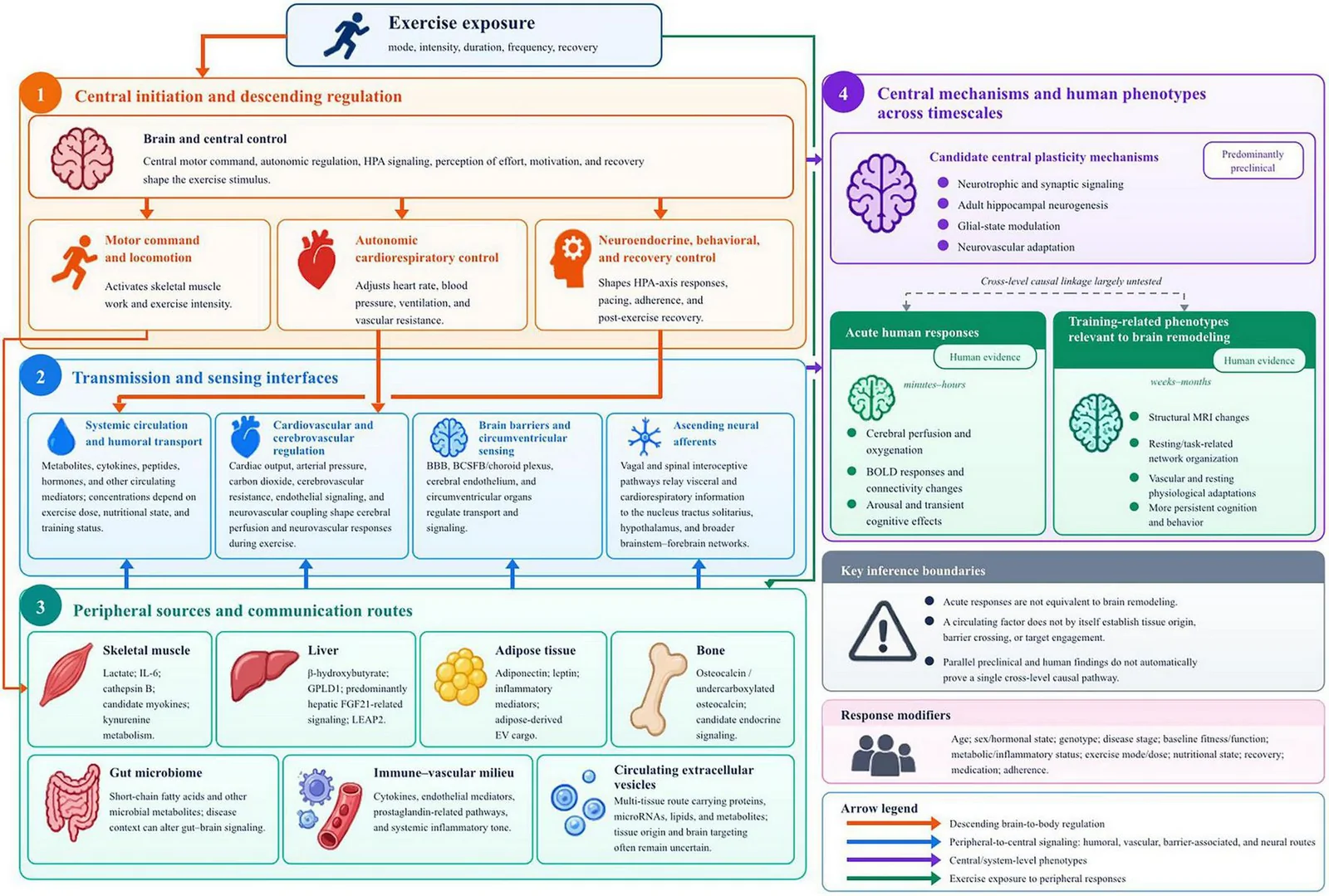
Integrative framework of bidirectional brain–body crosstalk in exercise-related brain remodeling. Exercise is represented as a bidirectional perturbation: central regulation shapes the physiological stimulus, while peripheral and systemic responses feed back to the brain. The four domains show (1) central initiation and descending regulation; (2) transmission and sensing interfaces; (3) peripheral sources and communication routes; and (4) candidate central mechanisms and human phenotypes across timescales. Orange arrows indicate descending regulation, blue arrows peripheral-to-central signaling, green arrows links from exercise exposure to peripheral responses, and purple arrows connections to central/system-level evidence domains. Peripheral-to-central routes include humoral transport, vascular regulation, barrier-associated sensing, and neural afferents. Candidate plasticity mechanisms, supported predominantly by preclinical evidence, are distinguished from acute human responses and training-related human phenotypes relevant to brain remodeling. Gray dashed links highlight largely untested cross-level causal linkage rather than an established sequence. Acute responses and imaging phenotypes alone do not establish cellular or molecular remodeling. Circulating factors do not by themselves establish tissue origin, barrier crossing, or target engagement, and parallel preclinical and human findings do not prove a single causal pathway. Response modifiers summarize factors that may alter these relationships.

A critical distinction in this framework is the separation of acute exercise responses from longer-term remodeling. Acute exercise can produce transient changes in lactate, cytokines, catecholamines, cerebral perfusion, arousal, neural activity, and cognitive performance over seconds to hours. These responses represent immediate regulatory states and candidate signals rather than evidence of durable brain adaptation. Repeated exercise exposure over weeks to months may instead produce more persistent changes in cardiorespiratory fitness, vascular function, metabolic capacity, synaptic regulation, neural circuitry, brain structure, and behavioral performance. Acute responses may contribute to chronic adaptation when they are repeatedly engaged, but this relationship cannot be assumed without temporally ordered or longitudinal evidence. Accordingly, exercise-responsive signals are interpreted throughout this review according to their timescale, biological source, and level of causal inference.

### 3.1 Central initiation and descending brain-to-body regulation during exercise

Exercise is not initiated solely by peripheral metabolic disturbance; rather, it represents a centrally coordinated physiological state involving descending motor, autonomic, neuroendocrine, and behavioral regulation. Before and during movement, central motor commands contribute to skeletal muscle activation while simultaneously coordinating cardiovascular and respiratory adjustments to anticipated metabolic demands. These feedforward signals interact continuously with peripheral feedback from muscle, arterial baroreceptors, chemoreceptors, and cardiopulmonary sensors, allowing cardiovascular and autonomic responses to be adjusted throughout exercise (Fisher et al., 2015; Wan et al., 2023).

The autonomic nervous system provides a major pathway through which the brain regulates peripheral physiological responses during exercise. Central command and peripheral reflexes interact to increase sympathetic outflow and modify parasympathetic activity, thereby influencing heart rate, cardiac output, vascular resistance, and blood pressure according to exercise intensity and metabolic demand (Fisher et al., 2015; Wan et al., 2023). Following exercise, parasympathetic reactivation contributes importantly to cardiovascular recovery and restoration of autonomic homeostasis, although its time course varies with exercise intensity, duration, and training status (Stanley et al., 2013). Autonomic regulation should therefore be considered an active component of descending brain-to-body control rather than merely a secondary consequence of peripheral metabolic change.

Neuroendocrine pathways provide an additional mechanism linking central regulation with peripheral responses. Exercise activates the hypothalamic–pituitary–adrenal (HPA) axis, with the magnitude of the response depending particularly on exercise intensity and duration. Acute activation increases circulating glucocorticoids and contributes to the coordinated metabolic and physiological stress response, whereas repeated exercise can modify HPA-axis responsiveness without necessarily producing chronically elevated basal cortisol concentrations (Duclos and Tabarin, 2016). The meaning of an exercise-related cortisol response therefore depends on workload, duration, training status, circadian timing, and recovery conditions rather than providing a simple marker of either benefit or physiological stress.

Central regulation also shapes exercise behavior. Perception of effort is a centrally integrated experience linked to motor command and other exercise-related sensory information and contributes to the regulation of exercise intensity and task continuation (Pageaux, 2016). Fatigue, effort perception, and motivational state may consequently influence subsequent exercise behavior and cumulative exposure, thereby modifying the repeated physiological stimulus from which longer-term adaptation emerges. This behavioral pathway should be distinguished from direct autonomic or endocrine efferent signaling, but it represents an additional route through which central processing can influence future brain–body interactions.

Together, these descending pathways establish the brain as an active regulator of exercise-induced systemic responses. Motor commands, autonomic outputs, neuroendocrine signaling, and behavior operate alongside ascending sensory and molecular feedback, providing the physiological basis for a genuinely bidirectional model of exercise-related brain–body communication.

### 3.2 Rapid signals from contracting skeletal muscle

Skeletal muscle is a major sensor of mechanical and energetic stress. Human studies show that endurance training modifies CaMKII expression and signaling, while acute endurance exercise increases the nuclear abundance of PGC-1alpha; AMPK can directly phosphorylate PGC-1alpha and coordinate metabolic adaptation (Rose et al., 2007; Little et al., 2010; Jäger et al., 2007). These observations establish intracellular exercise sensing, but they do not by themselves demonstrate secretion into the circulation or action on the brain.

Several exercise-responsive molecules have nevertheless become prominent candidates for muscle-to-brain communication. Contracting muscle contributes to the exercise-related rise in interleukin-6, which participates in substrate mobilization and post-exercise immune regulation in a source-, concentration-, and time-dependent manner (Xu et al., 2025). Lactate is both a metabolic substrate and a signaling molecule; it can enter the brain through monocarboxylate transport systems and has been linked experimentally to receptor-mediated and epigenetic processes, although much of the mechanistic evidence remains preclinical (Zhu et al., 2025; Abbott et al., 2010; Dienel and Hertz, 2005). Circulating irisin may change with exercise intensity, but estimates depend strongly on assay performance, training status, and sampling time (Huh et al., 2012; Daskalopoulou et al., 2014). Running-induced cathepsin B has been associated with memory-related outcomes across species, yet the human component does not establish causal mediation (Moon et al., 2016). Interleukin-15 and insulin-like growth factor 1 also respond variably to exercise mode and dose (Rodríguez-Gutiérrez et al., 2023; Duan et al., 2024; Khalafi et al., 2024). The skeletal-muscle PGC-1alpha1-kynurenine aminotransferase pathway illustrates the importance of species and context: animal work supports conversion of kynurenine toward less blood-brain-barrier-permeable kynurenic acid, whereas human training studies have not consistently demonstrated parallel changes in circulating metabolites (Agudelo et al., 2014; Allison et al., 2019).

A critical distinction concerns local versus endocrine action. Muscle-derived brain-derived neurotrophic factor appears well suited to local autocrine or paracrine functions at neuromuscular sites, whereas its contribution to circulating BDNF is limited and difficult to isolate (Zhang et al., 2025; Severinsen and Pedersen, 2020). The term exerkine is therefore useful only when source, release, exposure, and target engagement are specified. A peripheral concentration change alone cannot identify the dominant tissue source or prove delivery to a neural target.

Disease-related muscle phenotypes may further modify this signaling environment. Sarcopenia, which frequently co-occurs with cognitive impairment and neurodegenerative disorders, has been linked to overlapping inflammatory, vascular, mitochondrial, and hormonal–metabolic abnormalities; however, much of the evidence supporting a bidirectional muscle–brain relationship remains associative or is inferred from parallel mechanistic literatures rather than direct source-to-target studies (Liu Y. et al., 2026). Thus, disease-related changes in muscle quantity, quality, and bioenergetic function should be considered potential modifiers of exercise-responsive muscle–brain signaling rather than established causal drivers of neurodegeneration.

### 3.3 Liver, adipose tissue, and bone as endocrine-metabolic partners

The liver coordinates glucose homeostasis, substrate redistribution, ketogenesis, and endocrine adaptation during exercise (Trefts et al., 2015). When exercise is prolonged, liver glycogen is reduced, or nutritional availability is low, beta-hydroxybutyrate may rise. Beyond serving as an alternative fuel, beta-hydroxybutyrate can inhibit histone deacetylases and participate in histone beta-hydroxybutyrylation (Shimazu et al., 2013; Xie et al., 2016). It can also regulate hepatic LEAP2 production in experimental systems (Holm et al., 2022). Whether these mechanisms directly mediate routine exercise effects on the human brain depends on nutritional state, exercise dose, transport kinetics, and target-cell exposure.

Exercise-related plasma factors from the liver have also attracted attention. In aged mice, glycosylphosphatidylinositol-specific phospholipase D1 was identified as a liver-derived factor capable of transferring part of the neurogenic and cognitive effects associated with exercise (Horowitz et al., 2020). Fibroblast growth factor 21 is another candidate linking energetic stress, systemic metabolism, and cerebrovascular protection, but its organ-specific contribution and human brain targets remain incompletely resolved (Li et al., 2023; Bieri et al., 2023; Wang et al., 2025).

Adipose tissue contributes adiponectin, leptin, inflammatory mediators, and potentially adipose-derived extracellular-vesicle cargo to systemic signaling. Training-related increases in adiponectin and decreases in leptin are often accompanied by changes in fat mass or energy balance, making it difficult to isolate direct exercise effects (Nurnazahiah et al., 2016). Leptin can engage saturable blood-to-brain transport and hypothalamic sensing, but transport efficiency and central resistance vary with metabolic state (Banks et al., 2000; Bonnavion et al., 2015). Adipose-derived extracellular vesicles represent a plausible additional route of inter-organ communication; their cellular and depot-specific origins are heterogeneous, and evidence for exercise-responsive adipose EV release, biodistribution, or direct neural target engagement remains limited (Yang et al., 2023). Accordingly, current evidence supports adipose tissue as a contributor to systemic metabolic signaling, whereas a direct exercise-induced adipose-to-brain causal pathway remains insufficiently established.

Bone is also an endocrine organ. Mechanical loading may alter osteocalcin and undercarboxylated osteocalcin, and animal studies support links with metabolism and cognition; however, the active molecular form, assay comparability, and causal significance in humans remain debated (Chen et al., 2024; Yuan and Song, 2022; Zanatta et al., 2014). These examples support a network view in which muscle, liver, adipose tissue, and bone provide partially overlapping signals whose relative importance changes with exercise mode, intensity, duration, nutritional state, and baseline health. Table 1 summarizes the principal candidate pathways and their limitations.

### 3.4 Gut microbiome, immune-vascular milieu, and extracellular vesicles

Evidence concerning the gut microbiome is strongest for repeated training and longer-term adaptation. Some studies report changes in short-chain-fatty-acid-producing taxa or in butyrate, propionate, and acetate, but human findings are heterogeneous and sensitive to diet, adiposity, medication, training status, geography, and sampling strategy (Zhang et al., 2025; Tang et al., 2024; Cerdá et al., 2016; Vazquez-Medina et al., 2024). Short-chain fatty acids can act as substrates and signaling molecules through G-protein-coupled receptors and histone-deacetylase-related pathways, influencing immune and enteroendocrine processes (Kim, 2023; Lund et al., 2018). Exercise may also modify bile-acid transformation and tryptophan or nitrogen metabolism, but the direction and clinical meaning of these changes are not consistent across studies (Tang et al., 2024; Aoi et al., 2023; Vazquez-Medina et al., 2024).

Disease context is similarly important for gut–brain communication. In Parkinson’s disease, gut dysbiosis has been linked to altered barrier integrity, systemic inflammation, microbial metabolites, and α-synuclein-related pathology, and recent fecal microbiota transplantation trials have tested whether modifying the microbiome can alter clinical outcomes. Results remain inconsistent: some trials report motor or gastrointestinal improvement, whereas others show no significant primary clinical benefit despite measurable microbiome shifts (Tan et al., 2026). These findings illustrate that perturbing the gut microbiome can provide mechanistic and translational information, but clinical response cannot yet be attributed to a specific microbial pathway or assumed to generalize across neurodegenerative diseases.

The immune-vascular milieu provides another route by which systemic exercise responses may influence the brain without requiring a molecule to enter the brain parenchyma. Cytokines and endothelial mediators can act on cerebral vascular receptors, alter prostaglandin-related signaling, and modify neurovascular or glial states (Konsman et al., 2004; Sun et al., 2025; da Luz Scheffer and Latini, 2020). Human blood markers, however, are not brain-specific and may integrate responses from many tissues. Their interpretation depends on timing, cellular source, plasma-volume changes, and the distinction between transient immune activation and chronic inflammatory regulation.

Extracellular vesicles can transport proteins, microRNAs, lipids, and metabolites, potentially increasing cargo stability and supporting long-distance communication (Yáñez-Mó et al., 2015; Wu et al., 2025). Their importance lies in packaging and delivery rather than in representing one independent organ signal. Exercise-associated changes in vesicle abundance or cargo are increasingly reported, but source tissue, biodistribution, interface crossing, target-cell uptake, and biologically effective dose remain major unresolved questions.

## 4 Hemodynamic regulation, transmission, and sensing at the peripheral-central interface

Communication between peripheral organs and the brain occurs through interacting hemodynamic, vascular, humoral, and neural pathways. Central autonomic and neuroendocrine outputs regulate cardiac function, vascular tone, ventilation, and systemic metabolism, whereas peripheral metabolic and sensory signals are conveyed back to the brain through circulation, vascular interfaces, barrier-associated signaling, and neural afferents. These pathways operate simultaneously and should not be interpreted as independent conduits. In particular, exercise-related changes in cerebral perfusion and blood-oxygen-level-dependent signals depend not only on neural activity but also on cardiac output, arterial pressure, arterial carbon dioxide tension, endothelial function, and cerebrovascular reactivity. The peripheral-central interface is therefore better understood as a dynamic regulatory system than as a passive barrier separating circulating signals from the brain.

### 4.1 Cardiovascular and cerebrovascular regulation

Exercise produces rapid cardiovascular adjustments that are centrally coordinated but continuously modified by peripheral feedback. Central command, the exercise pressor reflex, arterial baroreflexes, and chemoreflexes interact through autonomic pathways to alter heart rate, cardiac output, vascular resistance, and arterial pressure, while local metabolic vasodilation redistributes blood flow toward active tissues (Fisher et al., 2015; Wan et al., 2023). Cardiovascular regulation therefore constitutes an active component of brain–body crosstalk rather than merely a background physiological response to exercise.

Cerebral perfusion is regulated within this broader cardiovascular environment but is not determined by cardiac output alone. Cerebral blood flow reflects the integrated influence of arterial carbon dioxide tension, cerebral metabolism, arterial pressure, neurogenic regulation, cardiac output, and cerebrovascular resistance (Smith and Ainslie, 2017). Changes in ventilation are particularly relevant because hyperventilation during strenuous exercise can reduce arterial carbon dioxide tension and produce cerebral vasoconstriction even while systemic cardiac output remains elevated. Consequently, an exercise-related increase or decrease in cerebral perfusion cannot be interpreted as a direct measure of neuronal activation or brain benefit without considering the accompanying respiratory and vascular state.

Endothelial function provides an additional link between repeated exercise and longer-term vascular adaptation. Recurrent changes in blood flow, shear stress, arterial pressure, and wall stress can induce functional and structural adaptation of the vasculature, with nitric-oxide-dependent signaling contributing to exercise-related endothelial responses (Green et al., 2017). Arterial stiffness is also relevant to brain health because greater central stiffness and pulsatility may increase the hemodynamic load transmitted to the cerebral circulation, although the extent to which exercise-induced changes in arterial stiffness directly mediate brain outcomes remains uncertain (Barnes et al., 2021). Cardiorespiratory fitness may therefore be viewed as an integrative phenotype associated with systemic and cerebrovascular function rather than as evidence of any single causal pathway.

A distinction between acute hemodynamic responses and chronic cerebrovascular adaptation is essential. Acute exercise can transiently alter cerebral blood flow, arterial pressure, carbon dioxide tension, vascular tone, and neurovascular coupling over seconds to minutes (Smith and Ainslie, 2017). Higher cardiorespiratory fitness is associated with lower cerebrovascular resistance and greater cerebrovascular reactivity, but intervention evidence for training-induced changes in resting middle cerebral artery velocity or global cerebral blood flow is limited and heterogeneous; improvements in cerebrovascular reactivity also remain less consistent than cross-sectional fitness associations (Smith et al., 2021). Acute hemodynamic responses should therefore be regarded as physiological states that may contribute to adaptation rather than as evidence of long-term brain remodeling in themselves.

This distinction is also important for interpretation of neuroimaging. Blood-oxygen-level-dependent functional MRI reflects changes in blood oxygenation arising from coupled neural, metabolic, and vascular processes rather than a direct measurement of neuronal activity (Ogawa et al., 1990). Exercise-related differences in BOLD amplitude, connectivity, or cerebral perfusion may therefore reflect changes in vascular reactivity or systemic physiology in addition to neural adaptation. Where possible, exercise-neuroimaging studies should account for cardiorespiratory fitness, arterial pressure, carbon dioxide regulation, and cerebrovascular reactivity when attributing imaging changes to neural plasticity.

### 4.2 Blood-brain barrier and neurovascular signaling

The blood-brain barrier is formed by specialized brain microvascular endothelial cells together with pericytes, basement membrane, astrocytic endfeet, and other components of the neurovascular unit (Andjelkovic et al., 2023; Abbott et al., 2010). Polar substrates such as glucose rely on transporters, while lactate and beta-hydroxybutyrate can use monocarboxylate transporter systems (Abbott et al., 2010; Mergenthaler et al., 2013; Dienel and Hertz, 2005). Large peptides and proteins generally do not diffuse freely. Experimental studies suggest that circulating insulin-like growth factor 1 may influence the brain through vascular or choroid-plexus-related mechanisms, but these findings cannot be assumed to reproduce human post-exercise kinetics (Carro et al., 2000, 2005; Pan and Kastin, 2000). Early animal work reported saturable transport of BDNF, yet the physiological contribution of peripheral BDNF to brain BDNF remains uncertain (Pan et al., 1998).

The barrier should also be viewed as a signaling platform. Peripheral inflammatory stimuli can activate receptors and cyclooxygenase-related pathways in brain vessels, generating central-side mediators without bulk entry of the original molecule (Konsman et al., 2004). Regular exercise may support barrier integrity in aging or disease models, but evidence is insufficient to conclude that exercise generally enhances selective entry of beneficial factors or normalizes specific transporters in healthy humans (Sun et al., 2025).

### 4.3 Blood-cerebrospinal fluid barrier and circumventricular sensing

The choroid plexus and blood-cerebrospinal fluid barrier have transport and signaling properties distinct from the blood-brain barrier (Reinhold and Rittner, 2017; Lun et al., 2015). Animal studies implicate the megalin/LRP2 system in serum insulin-like growth factor 1 signaling through the choroid plexus, but the spatial and temporal distribution of exercise-responsive IGF-1 in cerebrospinal fluid and brain tissue remains unclear (Carro et al., 2000, 2005). Irisin concentrations in cerebrospinal fluid have been associated with disease-related markers, but such associations do not demonstrate exercise-specific transport or a defined route across the choroid plexus (Dicarlo et al., 2024).

Circumventricular organs contain fenestrated capillaries and specialized cellular boundaries that support sensing of osmotic, hormonal, and inflammatory signals (Miyata, 2022; Bentivoglio et al., 2018; Kiecker, 2018). The organum vasculosum of the lamina terminalis, subfornical organ, and area postrema are prominent sensory structures, whereas the median eminence has a major neuroendocrine exchange function. Direct evidence that an exercise-elevated myokine or lactate signal is rapidly detected by a specific circumventricular organ and then transmitted to a hippocampal target is lacking. These structures are better considered parallel sensing interfaces that can influence hypothalamic and brainstem regulation than proven conduits for individual exerkines.

### 4.4 Ascending neural afferent pathways and brainstem-hypothalamic integration

Vagal afferents provide a rapid visceral-to-brainstem channel, while spinal interoceptive pathways and humoral routes operate in parallel (Münzberg et al., 2023; Luo et al., 2025). During exercise, changes in heart rate, ventilation, gastrointestinal and hepatic perfusion, and immune state occur alongside dynamic autonomic adjustment (Shanks and Ramchandra, 2025). Not every metabolic or inflammatory signal is directly encoded by vagal terminals, and the specificity of exercise-related afferent coding remains incompletely characterized.

Vagal afferents project primarily to the nucleus tractus solitarius, which distributes information to hypothalamic, limbic, noradrenergic, serotonergic, and forebrain networks. The locus coeruleus and dorsal raphe are widespread neuromodulatory systems rather than equivalent first-order relays, and exercise-related monoaminergic responses vary by brain region, time, and workload (Lopez Blanco and Tyler, 2025; Huang et al., 2025; Nicastro and Greenwood, 2016). The hypothalamus integrates metabolic, hormonal, circadian, and autonomic information and is therefore a major hub linking peripheral homeostasis with central output (Mendoza-Viveros et al., 2023; Bonnavion et al., 2015). Its direct mediation of cognitive benefit from a given exercise prescription, however, requires region-specific and longitudinal evidence.

## 5 Candidate mechanisms of exercise-related central plasticity

Signals detected through vascular, barrier-associated, humoral, and neural pathways can interact with central motor activity, autonomic regulation, and exercise-related hemodynamic changes. However, the detection of an acute signal does not establish long-term neural remodeling. In this section, molecular and cellular findings are considered candidate mechanisms of exercise-related plasticity only when they provide evidence relevant to sustained changes in gene regulation, synaptic organization, cellular state, neurogenesis, or neurovascular adaptation. Much of this evidence derives from animal or cellular models and should therefore be distinguished from direct evidence of persistent adaptation in humans.

### 5.1 Neurotrophic and synaptic signaling

Locally produced BDNF binds tropomyosin receptor kinase B and activates MAPK/ERK-CREB, PI3K-Akt-mTOR, and PLC-gamma pathways that regulate gene expression, survival, local translation, calcium signaling, and several forms of synaptic plasticity (Cefis et al., 2023; Lu et al., 2023; Liu et al., 2025). Animal studies report exercise-related changes in TrkB expression or phosphorylation and interactions with NMDA receptors, CaMKII, AMPA receptors, cytoskeletal pathways, dendritic complexity, spine density, and synapsin I (Jemni et al., 2023; Vaynman et al., 2003, 2004; Tong et al., 2025; Li et al., 2017; Shih et al., 2013; Stranahan et al., 2007). These findings establish strong biological plausibility for exercise-sensitive synaptic remodeling in experimental models.

The inferential boundary is important. A change in circulating BDNF does not identify brain BDNF production, and a molecular change in an animal hippocampus is not equivalent to demonstrated synaptic remodeling in a living human. Human studies need repeated, temporally ordered measurements and target-engagement indicators capable of testing whether a proposed pathway mediates changes in behavior rather than merely covaries with them.

### 5.2 Adult hippocampal neurogenesis, glial states, and neurovascular adaptation

Exercise-induced adult hippocampal neurogenesis is one of the most reproducible findings in rodents, involving precursor proliferation, survival, maturation, and circuit integration, with BDNF, insulin-like growth factor 1, and vascular endothelial growth factor among the candidate regulators (Reddy et al., 2023; Zhao, 2024; Farmand et al., 2025). The human situation is more uncertain. Studies have reported both very low or undetectable adult neurogenesis and persistent neurogenic cell populations in healthy adult and aging brains, with reductions in Alzheimer disease; recent single-cell and tissue evidence has renewed the debate (Sorrells et al., 2018; Boldrini et al., 2018; Moreno-Jiménez et al., 2019; Disouky et al., 2026). Even if adult human neurogenesis occurs, its magnitude, functional contribution, and responsiveness to exercise in living humans remain unestablished. It should therefore be presented as a cross-species candidate mechanism, not as a proven basis for clinical exercise prescription.

Experimental studies indicate that exercise can alter astrocytic, microglial, and ependymal responses, but these findings arise predominantly from animal and cellular models, and glial states cannot be reduced to a simple pro-inflammatory versus anti-inflammatory binary. Phenotypes depend on cell type, brain region, age, disease context, and exercise dose (Liu R. et al., 2026; Maugeri et al., 2024). Experimental work further indicates that lactate receptor HCAR1 signaling can induce cerebral vascular endothelial growth factor expression and angiogenesis (Morland et al., 2017). These findings support the biological plausibility of neurovascular adaptation, but they do not demonstrate that the same pathway mediates persistent brain remodeling or cognitive benefit in humans.

## 6 Human systems-level phenotypes: acute responses and training-related adaptations

Human neuroimaging and behavioral studies provide a complementary level of evidence to the predominantly preclinical mechanisms discussed above. Rather than directly measuring molecular or cellular remodeling, these studies capture systems-level phenotypes across different timescales, including cerebral perfusion, structural and functional imaging measures, and cognitive or behavioral outcomes. Acute studies primarily characterize transient changes in cerebral perfusion, BOLD responses, connectivity, arousal, or task performance during or shortly after a single exercise bout, whereas longitudinal training studies examine whether repeated exercise is associated with more persistent alterations in brain structure, resting physiology, network organization, or cognition. These forms of evidence should not be treated as interchangeable: acute responsiveness may identify a physiological state or candidate pathway relevant to adaptation, but neither acute nor longitudinal systems-level measures alone can establish the molecular or cellular mechanism responsible for an observed human effect.

### 6.1 Structural imaging

Longitudinal magnetic resonance imaging studies provide some of the clearest human evidence for training-related changes in macroscopic brain structure, although populations, interventions, sample sizes, and analytic pipelines are heterogeneous (Xu et al., 2024). A landmark randomized trial in older adults reported an approximately 2% mean increase in hippocampal volume after 1 year of moderate-intensity aerobic training, while an earlier 6-month intervention found volume increases in selected frontal and temporal regions (Erickson et al., 2011; Colcombe et al., 2006). These studies support the plasticity of macroscopic structural measures, but MRI volume and cortical thickness reflect mixtures of cellular, vascular, extracellular-space, and water-related processes.

Diffusion MRI measures such as fractional anisotropy and mean diffusivity are sensitive to tissue microstructure but lack cellular or pathological specificity. Crossing fibers, extracellular water, axonal organization, and myelin can all influence the signal (Jones et al., 2013). Associations between physical activity or fitness and white-matter tracts, as well as skill-related cerebellar differences, are therefore informative phenotypes rather than direct measurements of axonal number, conduction speed, or network stability (Bashir et al., 2021; Roberts et al., 2013; Piervincenzi et al., 2017).

### 6.2 Functional imaging: acute state changes versus longer-term network adaptation

Blood-oxygen-level-dependent functional MRI provides an indirect hemodynamic signal that is sensitive to neural activity (Ogawa et al., 1990). Resting-state research established low-frequency synchronization in the absence of an explicit task and subsequently identified large-scale intrinsic networks (Biswal et al., 1995). Importantly, functional imaging findings should be interpreted according to the timescale of exercise exposure. Acute exercise may produce transient changes in BOLD responses, cerebral perfusion, connectivity, or cortical oxygenation, whereas repeated training may be associated with more persistent changes in network organization. Randomized exercise interventions and observational studies report changes in default-mode and executive-control connectivity and associations with memory (Voss et al., 2010; Sohn et al., 2025; Lloyd et al., 2024). Functional near-infrared spectroscopy studies also report altered prefrontal oxygenation and activation patterns after exercise (Cai and Li, 2026). Acute imaging responses should therefore be considered exercise-responsive physiological states rather than direct evidence of long-term brain remodeling.

These findings demonstrate that network measures are exercise-sensitive, not that a particular network change is the causal mediator of benefit. BOLD signals integrate vascular and neural influences, and many studies lack repeated temporal measurements, active controls, formal mediation analyses, or external replication. Athlete studies often invoke neural efficiency, but predominantly cross-sectional designs cannot exclude selection, long training histories, or lifestyle differences (Li and Smith, 2021; Guo et al., 2017; Yan et al., 2025). Resting-state differences, task-evoked compensation, and greater or lower activation are not interchangeable constructs.

### 6.3 Cognition and behavior across acute and training timescales

Cognitive and behavioral outcomes are the system-level phenomena that an integrative brain-body framework must ultimately explain, but acute responses and longer-term training effects should be distinguished. Meta-analyses generally support small-to-moderate average effects of exercise on executive function and other domains, with substantial variation by age, intervention duration, comparison condition, test selection, and risk of bias (Ye et al., 2024; Zang et al., 2024; Ludyga et al., 2023). Training studies address whether repeated exercise produces more persistent cognitive adaptation; for example, 1-year aerobic training has been linked with hippocampal volume and spatial memory (Erickson et al., 2011). In contrast, acute studies examine transient changes during or shortly after a single exercise bout, including dentate-gyrus-related pattern separation and hippocampal connectivity, motor-sequence memory, and selective attention (Suwabe et al., 2018; Marin Bosch et al., 2020; Ligeza et al., 2023). Other work has examined executive-related oculomotor control in physically active individuals (Zhou and Zhuang, 2023). These outcomes are not interchangeable, and an immediate post-exercise cognitive change should not be interpreted as evidence of persistent cognitive remodeling.

The human intervention literature is also less uniformly positive than pooled mean effects may suggest. Clinical trials in populations with mild cognitive impairment or dementia have reported modest, inconsistent, or null cognitive effects despite measurable changes in fitness or other physiological outcomes (Baker et al., 2025; Lamb et al., 2018). Estimates can also depend on the comparator: active control conditions may attenuate apparent exercise effects relative to passive or usual-care controls, indicating that nonspecific intervention components can contribute to observed between-group differences (Ding et al., 2025). Repeated cognitive testing introduces additional practice effects, making between-group comparisons preferable to within-group improvement alone when the same or closely related tasks are administered repeatedly (Hindin and Zelinski, 2012). Interpretation is further complicated by the frequent assessment of multiple cognitive domains, tests, and derived outcomes within individual studies, which can increase analytical flexibility when outcomes are not prespecified. Publication bias also remains a concern: a recent three-level meta-analysis of randomized trials in cognitively healthy older adults reported small improvements in executive function but detected evidence of potential publication bias (Chen et al., 2026).

Behavioral improvement does not prove mediation by adult neurogenesis, white-matter conduction, or a specific monoaminergic pathway. The most defensible conclusion is therefore that acute exercise can transiently influence selected cognitive processes, whereas repeated exercise may produce more persistent benefits in some cognitive domains and populations. However, the magnitude and robustness of these effects depend on study design, comparator condition, outcome selection, population characteristics, and timescale, and neither acute nor training-related behavioral improvement by itself establishes the underlying biological mechanism.

## 7 Sources of response heterogeneity

Group-average effects do not imply uniform individual response. Biological characteristics, disease stage, exercise delivery, adherence, recovery, measurement error, and statistical power all influence observable outcomes. Heterogeneity should not be treated only as noise; it may reveal when a mechanism is active, when an intervention is tolerated, and which outcomes are responsive.

Baseline disease phenotypes may therefore reshape the brain–body signaling environment before an exercise stimulus is introduced. Sarcopenia may co-occur with inflammatory, vascular, mitochondrial, and metabolic abnormalities that also track cognitive decline, while neurodegenerative disorders such as Parkinson’s disease can involve gut dysbiosis, altered barrier function, medication effects, and disease-stage heterogeneity (Liu Y. et al., 2026; Tan et al., 2026). Such conditions can modify both the peripheral signals generated by exercise and the central systems available to respond, reinforcing the need to interpret exercise effects within disease-specific physiological contexts.

Genetic background is one candidate modifier. The BDNF Val66Met polymorphism can affect BDNF processing and activity-dependent secretion, and some studies report genotype-related differences in peripheral BDNF or neurobiological responses to exercise. Results are inconsistent, however, and peripheral BDNF is not a proxy for brain BDNF; the evidence does not justify genotype-based exercise prescription (Kambestad et al., 2023; de Las Heras et al., 2022; Ieraci et al., 2016). APOE epsilon4 may modify associations between physical activity and brain structure or function, but most evidence comes from observational studies, subgroup analyses, or modest samples (Smith et al., 2011, 2014; De Marco et al., 2015; de Frutos-Lucas et al., 2020).

Age, sex and hormonal state, baseline fitness, metabolic and inflammatory status, cognitive level, and disease burden may also influence response (Festa et al., 2023; Barros-Aragão et al., 2025; Dong et al., 2025; Stillman et al., 2016; Wrigglesworth et al., 2023). Higher baseline performance can produce ceiling effects, whereas lower baseline function can permit greater improvement but may also reflect greater disease burden or regression to the mean. Exercise mode and dose add another layer. Aerobic, resistance, skill-based, coordination, and mind-body exercise combine cardiorespiratory, metabolic, motor-learning, and emotional pathways in different proportions, but current human evidence does not support a one-to-one mapping between an exercise category and a single brain mechanism (Herold et al., 2019; Jahani et al., 2025; Tsai et al., 2015; Guo et al., 2017; Surkar et al., 2025; Zhang et al., 2022; Dhahbi et al., 2025; Li et al., 2025). Frequency, intensity, time, type, volume, progression, recovery, adherence, and baseline state should be modeled jointly rather than reduced to a single total-dose label.

## 8 Discussion

The central argument of this review is that exercise-related brain remodeling is best understood as a bidirectional process operating across interacting biological levels. Exercise engages central command, sensory feedback, autonomic regulation, cerebral hemodynamics, and peripheral organ responses at the same time. Skeletal muscle, liver, adipose tissue, bone, the gut microbiome, and the immune-vascular milieu generate candidate signals, but their significance depends on source, timing, interface engagement, target cell, and effective dose. The blood-brain barrier, blood-cerebrospinal fluid barrier, cerebral endothelium, circumventricular organs, and neural afferents are not interchangeable conduits. They are parallel sensing and transmission systems with different physiological constraints.

The evidence is strongest within several individual levels. Experimental models provide robust support for exercise-sensitive intracellular signaling, synaptic plasticity, adult hippocampal neurogenesis, glial modulation, and neurovascular adaptation. Human studies provide direct evidence that exercise can alter circulating biomarkers, cardiorespiratory fitness, cerebral perfusion, imaging phenotypes, and selected cognitive outcomes. The weakest part of the evidence base is the bridge across levels. Few human studies demonstrate, in temporal order, that an organ-specific signal reaches or activates a defined interface, engages a particular neural cell population, produces a specific imaging change, and thereby causes cognitive benefit. Table 2 makes this distinction explicit.

**TABLE 2:** Key controversies, evidence gaps, and priority research designs.

Controversy or inference problem	What current evidence supports	What remains unresolved	Why present human evidence is insufficient	Priority research design
Does peripheral BDNF mediate exercise-related brain effects?	Brain-derived BDNF-TrkB signaling is biologically important, and exercise changes BDNF-related measures in experimental studies.	The physiological contribution of peripheral BDNF to brain BDNF-TrkB signaling is unknown.	Circulating BDNF has mixed cellular sources, pre-analytical variability, and does not directly represent brain BDNF.	Source-resolved sampling, validated assays, repeated time-course measurements, and pathway-targeted intervention or mediation studies.
Does exercise enhance adult human hippocampal neurogenesis?	Exercise robustly enhances adult hippocampal neurogenesis in rodents, and adult human neurogenesis has renewed cellular support.	Magnitude, functional contribution, and exercise responsiveness in living humans are not established.	Human tissue studies are indirect for exercise exposure, whereas MRI cannot quantify new neurons.	Cross-species studies linking standardized exercise to molecular imaging, fluid markers, single-cell evidence, and tissue validation where feasible.
Do MRI volume or diffusion changes identify a specific cellular process?	Randomized and longitudinal studies show that selected structural MRI measures can change after training.	The relative contributions of vascular, glial, extracellular-space, water, myelin, axonal, synaptic, or neurogenic changes are unknown.	Volume, cortical thickness, fractional anisotropy, and mean diffusivity lack cell-type and process specificity.	Multimodal MRI combined with perfusion, spectroscopy, positron-emission tomography, electrophysiology, fluid biomarkers, and repeated behavior.
Is altered functional connectivity a causal mediator of cognitive benefit?	Interventions and observational studies report changes in resting-state and task-related network measures.	Connectivity changes may cause, compensate for, or merely accompany cognitive change.	BOLD signals are hemodynamic and correlational; many studies lack temporal mediation tests and active controls.	Preregistered longitudinal trials with repeated imaging, active comparators, temporal mediation, and out-of-sample replication.
Are exercise-related cognitive benefits robust across trials and outcome domains?	Meta-analyses indicate small average benefits in selected cognitive domains, and some longitudinal trials report improvement.	Magnitude and durability vary by population, comparator, cognitive domain, and timescale; null or modest effects are reported in some clinical trials.	Active controls can attenuate apparent effects; repeated testing introduces practice effects; multiple outcomes increase interpretive flexibility; publication bias may influence pooled estimates.	Preregistered randomized trials with active comparators, prespecified primary cognitive outcomes, strategies to limit practice effects, complete outcome reporting, and longer-term follow-up.
Does exercise-induced microbiome change mediate brain benefit?	Exercise can alter microbial composition and metabolites, and gut-derived molecules can influence immune and neural signaling.	Directionality, durability, and the fraction of brain or cognitive effects attributable to the microbiome remain unresolved.	Diet, adiposity, medication, geography, and baseline microbiome structure strongly confound associations.	Diet-controlled trials, dense longitudinal sampling, metagenomics-metabolomics integration, and mechanistic perturbation or transfer studies.
Can organ-specific signals be traced from source to brain target?	Multiple exerkines, metabolites, vesicles, and neural routes are biologically plausible and experimentally supported.	For many signals, the dominant source, route, target cell, and effective concentration are unknown.	Peripheral concentration changes alone cannot establish tissue origin, interface crossing, or target engagement.	Stable-isotope or molecular tracing, organ-specific perturbation, vesicle barcoding, interface sampling, and target-engagement biomarkers.
Can mechanistic biomarkers support individualized exercise prescription?	Age, sex and hormonal state, genotype, disease stage, baseline function, dose, and adherence modify observed responses.	No single biomarker or genotype has established clinical utility for routine prescription.	Most subgroup findings are underpowered and not externally validated; prediction models are prone to overfitting.	Large preregistered multicenter trials with prespecified interactions, calibration, external validation, decision-curve analysis, and safety outcomes.

The table distinguishes evidence within individual biological levels from evidence that directly connects levels in a causal chain. Proposed study designs are priorities rather than minimum requirements for every research question.

Several recurring controversies follow from this inferential gap. Peripheral BDNF is often discussed as though it represents central BDNF activity, despite uncertain source and transport. Adult hippocampal neurogenesis is sometimes used to explain human cognitive benefit even though exercise responsiveness cannot be measured directly in living humans. MRI volume, cortical thickness, fractional anisotropy, and functional connectivity are sometimes assigned cellular meanings that their signals cannot uniquely support. Microbiome associations are vulnerable to diet, medication, adiposity, geography, and reverse causation. Similar caution applies to extracellular vesicles and exerkines when tissue source and target engagement have not been established.

A more decisive research strategy should prioritize bridges rather than adding isolated measurements. First, source attribution requires stable-isotope or molecular tracing, organ-specific perturbation, validated assays, and dense time-course sampling. Second, interface engagement should be evaluated through transport kinetics, endothelial or choroid-plexus markers, cerebrospinal-fluid sampling when justified, and neural-afferent physiology. Third, human target engagement needs multimodal combinations of molecular imaging, perfusion, spectroscopy, electrophysiology, fluid biomarkers, and repeated behavioral assessment. Fourth, mechanistic claims require temporal mediation, active comparison groups, preregistration, and replication.

Cross-species translation may also be strengthened by defining exercise dose according to comparable physiological intensity domains rather than absolute treadmill speed, running distance, or session duration. Recent work in rats has demonstrated that the lactate threshold (LT) and gas-exchange threshold (GET) can be identified relative to maximal oxygen uptake and can distinguish training performed below versus above the moderate-to-heavy intensity boundary; importantly, training above, but not below, the LT produced corresponding adaptations in aerobic capacity and physiological thresholds (Inoue et al., 2025). Related exercise-neuroscience models have used VO2max-based intensity categories together with blood lactate and oxygen-consumption measurements to characterize intensity-dependent hippocampal responses, illustrating how physiological anchors used in humans can inform preclinical brain studies (Hiraga et al., 2025). More generally, animal exercise protocols should report and, where feasible, normalize exercise intensity to individual physiological capacity, together with modality, duration, frequency, incline, training status, habituation procedures, and relevant environmental and sampling conditions (Poole et al., 2020). However, threshold-based standardization should not be assumed to eliminate species- or protocol-related variability: lactate responses and maximal-lactate-steady-state determination can vary substantially across mouse strains and can be influenced by handling or aversive stimulation (Lønbro et al., 2019). Physiological harmonization can therefore improve the comparability of animal and human exercise exposures, but it does not by itself establish homologous molecular or neural mechanisms; temporal alignment, target-engagement measures, and matched biological outcomes remain necessary for cross-species causal inference.

Mechanism-informed precision exercise should remain a research objective rather than a current clinical promise. Age, disease stage, functional limitation, baseline fitness, medication, fall risk, preference, access, and adherence can already support pragmatic stratification. In contrast, no single circulating factor, genotype, microbiome profile, or imaging marker has demonstrated sufficient clinical utility for routine individualized prescription. Prediction models should be evaluated against simple baselines and should report calibration, external validation, interpretability, and potential net benefit. A community-based pilot trial, for example, suggested a dose-response relationship for aerobic exercise and fitness while cognitive outcomes were smaller and less consistent (Vidoni et al., 2015). Clinical trials in mild cognitive impairment and dementia also warn against promising disease modification from mechanistic plausibility alone (Baker et al., 2025; Lamb et al., 2018). In Parkinson disease, exercise recommendations appropriately emphasize aerobic, resistance, balance, gait, and task-specific goals together with safety and function (Osborne et al., 2022).

This review has limitations. It is narrative rather than systematic, and its literature selection and qualitative evidence labels are not equivalent to an exhaustive search or formal certainty assessment. The breadth of the framework necessarily compresses organ-specific and disease-specific details. Because this was a narrative integrative review, the selection process was not designed to achieve exhaustive study enumeration. Therefore, omission of a specific study should not be interpreted as evidence of absence, and the evidence characterizations should not be considered formal certainty ratings.

## 9 Conclusion

Exercise-related brain remodeling emerges from interacting central and peripheral processes rather than a single circulating molecule or a fully validated linear pathway. Candidate signals from muscle, liver, adipose tissue, bone, the gut, and the immune-vascular system may act through barrier transport, vascular and choroid-plexus signaling, circumventricular sensing, and neural afferents. These inputs interact with neurotrophic, synaptic, glial, neurogenic, and neurovascular responses and may correspond to macroscopic imaging and cognitive phenotypes. The strength of evidence is uneven: cellular and animal mechanisms are comparatively rich, whereas direct human mechanism and causal mediation across biological levels remain limited. Progress will depend on preregistered longitudinal interventions, source-resolved biomarkers, cross-species pathway validation, multimodal target-engagement measures, and externally validated models of response heterogeneity.
